# Ancillary studies on cell blocks from fine needle aspiration specimens of salivary gland lesions: A multi‐institutional study

**DOI:** 10.1002/dc.24939

**Published:** 2022-01-29

**Authors:** Seena Tabibi, Matthew Gabrielson, Carla Saoud, Katelynn Davis, Sintawat Wangsiricharoen, Ryan Lu, Isabella Tondi Resta, Kartik Viswanathan, William C. Faquin, Zubair Baloch, Zahra Maleki

**Affiliations:** ^1^ Department of Pathology The Johns Hopkins University School of Medicine Baltimore Maryland USA; ^2^ Department of Pathology and Laboratory Medicine, Hospital of the University of Pennsylvania University of Pennsylvania Philadelphia Pennsylvania USA; ^3^ Department of Pathology Emory University Hospital Midtown Atlanta Georgia USA; ^4^ Department of Pathology Massachusetts General Hospital and Harvard Medical School Boston Massachusetts USA

**Keywords:** ancillary studies, cell block, fine‐needle aspiration, histochemistry stains, immunohistochemistry, in situ hybridization, Milan System for Reporting Cytology, salivary gland

## Abstract

**Background:**

Ancillary studies are commonly performed on cell blocks prepared from fine‐needle aspiration (FNA) specimens. There are limited studies in application of ancillary studies on cell blocks from salivary gland (SG) FNAs. This multi‐institutional study evaluates the role of ancillary studies performed on cell blocks in the diagnosis of SG lesions, and their impact on clinical management.

**Method:**

The electronic pathology archives of three large academic institutions were searched for SG FNAs with ancillary studies performed on cell blocks. The patient demographics, FNA site, cytologic diagnosis, ancillary studies, and surgical follow‐up were recorded. If needed, the cytologic diagnoses were reclassified as per the Milan System for Reporting Salivary Gland Cytopathology (MSRSGC).

**Results:**

117 SG FNA cases were identified including 3, 10, 11, 6, 23, 4, and 60 cases in MSRSGC categories I, II, III, IVa, IVb, V, VI, respectively with surgical follow‐up available ranging from 27% to 100% within each category. Ancillary studies including histochemistry, immunocytochemistry (IHC), and in situ hybridization (ISH) were beneficial in 60%–100% of cases in each category. Risk of malignancy was 100% in both the suspicious for malignancy (V) and malignant (VI) categories. Ancillary studies improved diagnosis in 60% of non‐neoplastic cases (II, 6/10), 100% of benign neoplasm cases (IVa, 6/6), and 98.3% of malignant cases (VI, 59/60).

**Conclusion:**

Judicious and case‐based ancillary studies performed on SG FNA cell blocks with sufficient material can improve the diagnostic yield by further characterization of the atypical/neoplastic cells, particularly in MSRSGC categories IVa‐VI.

## 1 INTRODUCTION

Fine‐needle aspiration (FNA) is a well‐accepted procedure to evaluate salivary gland lesions.^1^, ^2^, ^3^, ^4^ It is up to 79% sensitive and 96% specific in detecting malignancy, and up to 96% sensitive and 98% specific in the detecting neoplasia, respectively.^5^ Although most commonly occurring salivary gland neoplasms pose little diagnostic challenge on FNA (i.e., pleomorphic adenoma or Warthin tumor), differentiating between non‐neoplastic processes, benign lesions, and/or malignancies is not always achievable on routine stains due to cellular heterogeneity and overlapping architectural features.^6^, ^7^

In an effort to standardize SG FNA reporting and streamline downstream clinical management, the Milan System for Reporting Salivary Gland Cytopathology (MSRSGC) established six distinct diagnostic categories with associated risk of malignancy (ROM) based on cytomorphologic features.^4^, ^8^, ^9^

In the era of precision diagnostics, ancillary studies are often being performed on cytology specimens to provide a specific diagnosis and even prognostic information for optimal patient management.^9^ A wide array of ancillary studies such as immunocytochemistry, fluorescence in situ hybridization (FISH), DNA or mRNA in situ hybridization (ISH) can be performed on cell blocks. Salivary gland neoplasia arises from a variety of cell types, which can be delineated utilizing immunocytochemistry. A small panel of immunostains may yield a definitive diagnosis, even with minimal material. For example, p16 a surrogate marker in diagnosing HPV‐related squamous‐cell carcinoma, allows for a more definitive diagnosis than cytological examination alone.^10^ However, cell blocks are not routinely prepared for all SG FNA cases due to utilization of aspirated material for direct microscopic examination and when cell blocks are available, they may be insufficient for ancillary studies.

In this multi‐institutional retrospective study, we evaluated the utility of cell blocks with subsequent performance of ancillary studies in the diagnosis of salivary gland lesions classified according to the MSRSGC.

## 2 MATERIALS AND METHODS

The study was conducted after obtaining institutional research approval in each institution. The electronic pathology archives of Massachusetts General Hospital (MGH), The Johns Hopkins hospital (JHH) (1999–2019), and Hospital of the University of Pennsylvania (HUP) (2015–2020) were retrospectively searched for FNAs of salivary glands with any ancillary studies performed on cell blocks. The inclusion criteria for case selection were all available salivary gland FNAs, in which a cell block was prepared and ancillary studies were performed. All cases had cell block slide(s) stained with the hematoxylin and eosin and additional ancillary studies. The cytology samples in this study were processed as Diff‐Quik stained on air‐dried slides, Pa stained alcohol‐fixative slides, or Thin‐Prep preparation of alcohol fixed aspirations.

Each institution reviewed its own cases individually and classified the cases into the MSRSGC categories. The ancillary studies included in this study were immunohistochemical stains, histochemical stain and stains for detection of mucin, bacterial, fungal and mycobacterial micro‐organisms and in situ hybridization. The following data points were recorded for each patient: tumor type, sex, age, biopsy site, FNA diagnosis, cytologic category per MSRSGC, type and results of ancillary studies performed, and surgical follow‐up diagnosis when available. The study included the pathology report review only.

## 3 RESULTS

**FIGURE 1:**
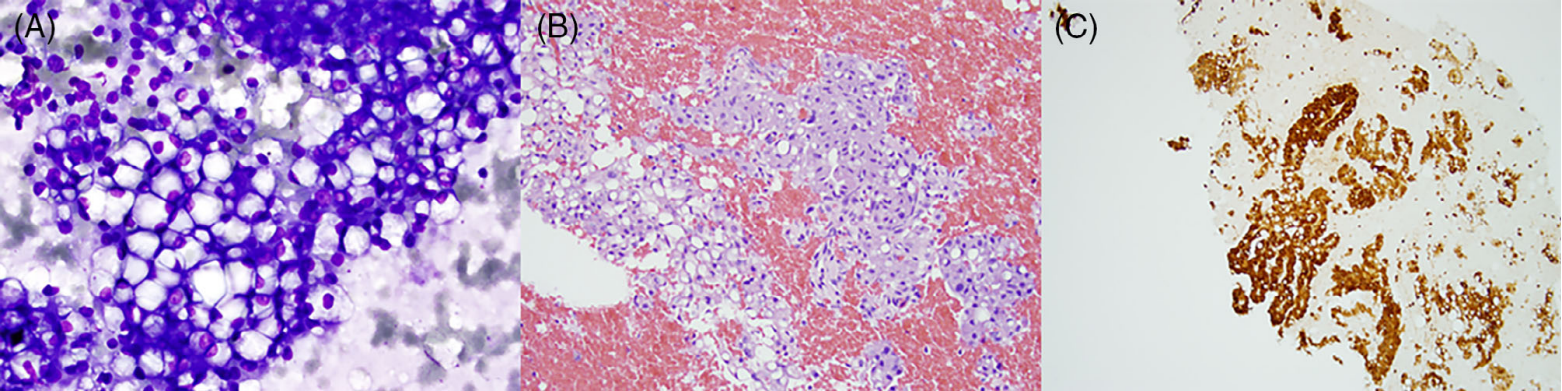
Secretory carcinoma, (A) A large fragment of cohesive cells is seen. The cells are characterized by large cytoplasmic vacuoles and round, uniform nuclei (×200, Diff‐Quik stain), (B) The cell block consists of large fragments of neoplastic cells containing abundant clear to eosinophilic cytoplasm (×200, H&E), (C) The tumor cells were positive for mammaglobin immunostain on cell block confirming the diagnosis (×200, Immunostain) [Colour figure can be viewed at wileyonlinelibrary.com]

**FIGURE 2:**
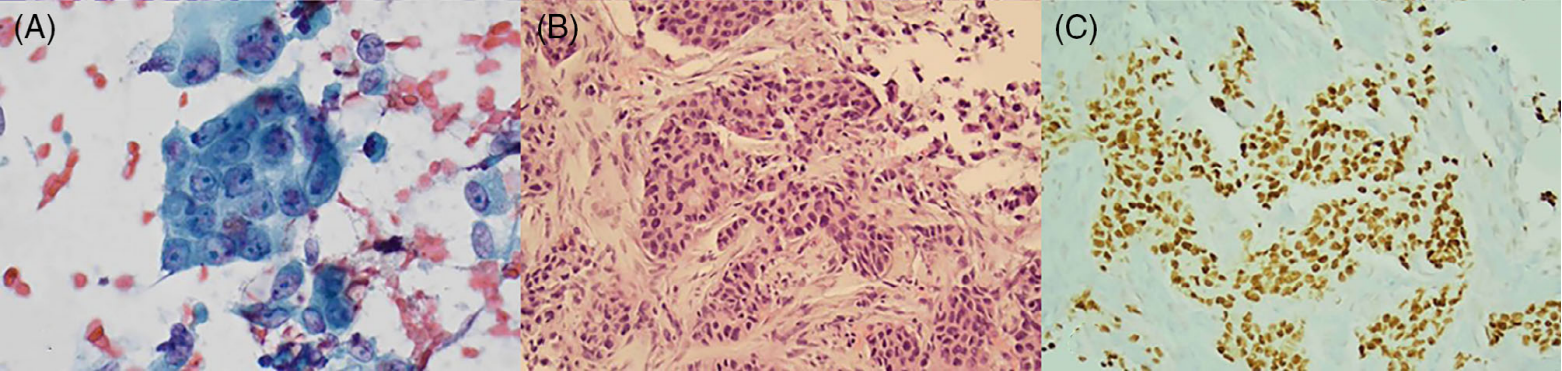
Salivary duct carcinoma, (A) Malignant epithelial cells are seen arranged in clusters and single cells. The nuclei exhibit anisonucleosis, thick nuclear membrane, course chromatin, and prominent nucleoli (×400, Papanicolaou stain). (B) A cell block showed infiltrating carcinoma, which could be primary or secondary based on morphology alone (×200, H&E). (C) The tumor cells expressed strong nuclear staining for androgen receptor immunostain on the cell block confirming salivary duct carcinoma (×200, immunostain) [Colour figure can be viewed at wileyonlinelibrary.com]

**FIGURE 3:**
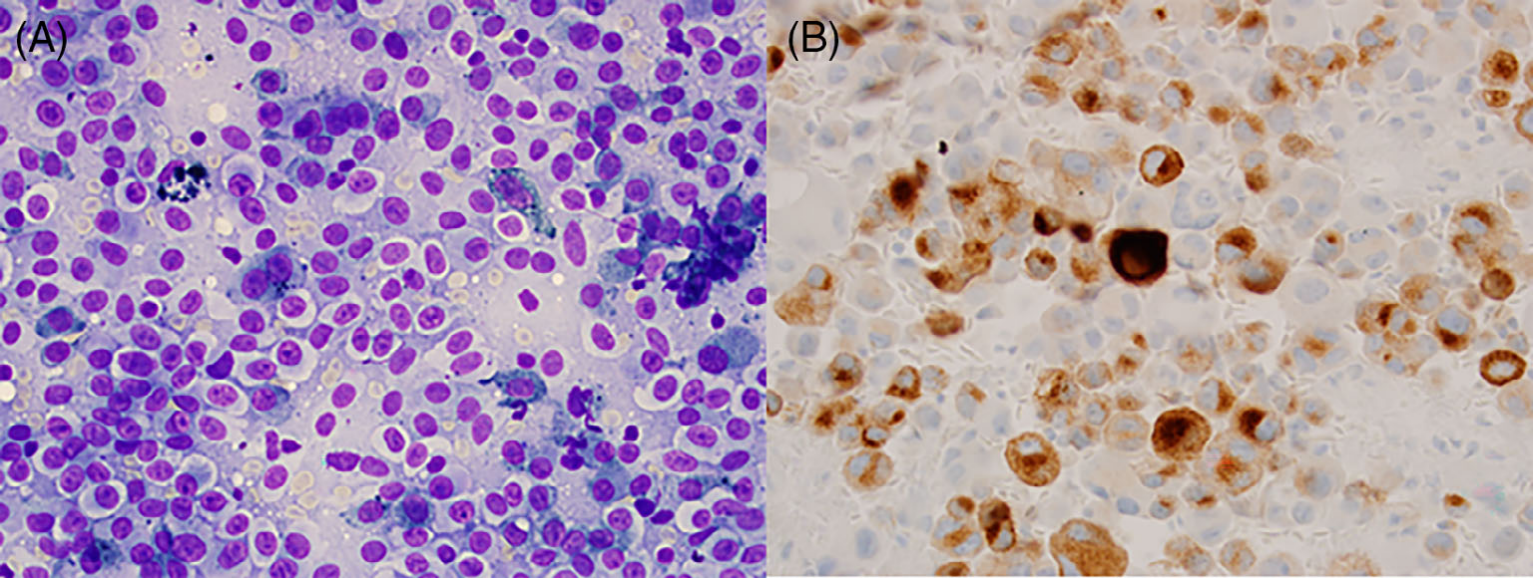
Malignant melanoma, (A) Numerous single cells are seen on a smear. The cells contain round to oval nuclei with moderate amount of cytoplasm. Occasionally cells contain melanin pigment (×200, Diff‐Quik). (B) The malignant cells were positive for Melan A on a cell block, confirming the diagnosis (×200, immunostain) [Colour figure can be viewed at wileyonlinelibrary.com]

One hundred and seventeen SG FNA specimens met the inclusion criteria. These included 67 male patients and 50 female patients, ranging in age from 2 to 92 years with a mean of 61.1 years and median of 63 years. The parotid gland was the most common site (101 lesions), followed by minor salivary glands (9 lesions), and submandibular gland (7 lesions). The MSRSGC diagnostic category distribution was as follows: 3 (2.6%) cases as non‐diagnostic, 10 (8.5%) as non‐neoplastic, 11 (9.4%) as atypia of undetermined significance (AUS), 6 (5.1%) as benign neoplasm, 23 (19.7%) as salivary gland neoplasm of uncertain malignant potential (SUMP), 4 (3.4%) as suspicious for malignancy, and 60 (51.3%) as malignant (Figures 1, 2, 3).

Tables 1, 2, 3, 4, 5, 6, 7 summarize cases according to MSRSGC category, cytology diagnosis before and after the ancillary study results, ancillary studies performed on the cell block and their results including the reason for performing ancillary studies, and surgical pathology diagnosis if available.

**TABLE 1:** MSRSGC category I: *non‐diagnostic*, cytology diagnosis, ancillary studies performed on cell block, and surgical pathology diagnosis (if applicable)

MSRSGC	FNA diagnosis without ancillary studies	Ancillary studies on cell block	FNA diagnosis with ancillary studies	Surgical pathology diagnosis	Reason for ancillary studies
I	Cyst contents with epithelioid cells	Positive for HAM56	Cyst contents with histiocytes	No surgical follow‐up	To rule out epithelial cells and identify macrophages
I	Cyst debris, mixed inflammation and epithelioid cells	Positive for CD68; Negative for AE1/AE3	Cyst debris, mixed inflammation with histiocytes	Cystic mucoepidermoid carcinoma	To rule out epithelial cells and identify macrophages
I	Salivary gland tissue with mixed inflammation	AE1/AE3 highlights normal salivary gland tissue; CD68 highlights histiocytes, and negative for S100, PAS; Ziehl Neelsen stain; Gram stain; and mucicarmine	Salivary gland tissue with mixed inflammation, no fungi, mycobacteria or bacteria identified	CLL	An infectious process is excluded

Abbreviation: CLL; chronic lymphocytic leukemia.

**TABLE 2:** MSRSGC category II: *non‐neoplastic*, cytology diagnosis, ancillary studies performed on cell block, and surgical pathology diagnosis (if applicable)

MSRSGC	FNA diagnosis without ancillary studies	Ancillary studies on cell block	FNA diagnosis with ancillary studies	Surgical pathology diagnosis	Reason for ancillary studies
II	Caseating granulomas	Positive Ziehl Neelsen stain	Caseating granulomas, mycobacterial organisms identified	No surgical follow‐up	Confirming mycobacteria organisms
II	Dense fibrosis with clusters of pigmented macrophages and scant benign salivary gland tissue	Negative for Iron stain	Dense fibrosis with clusters of pigmented macrophages (negative for Iron stain) and scant benign salivary gland tissue	No surgical follow‐up	Ruling out hemosiderin pigment
II	Chronic sialadenitis	Negative for IgG4	Chronic sialadenitis, negative for IgG4	No surgical follow‐up	Ruling out IgG4 related disease
II	Polymorphous lymphoid tissue with atypical lymphocytes, cannot exclude a lymphoproliferative disorder	Mix of CD3 positive T‐cells and CD20 positive B‐cells. AE1/AE3 highlights epidermis	Polymorphous lymphoid tissue	Benign lymph node with follicular hyperplasia	Ruling out lymphoma
II	Chronic inflammation and plasmacytosis, cannot rule out a plasma cell proliferative disorder	CD3 and CD20 highlight mixed population of T‐ and B‐Cells, respectively. C138 shows prominent plasma cell population that are polytypic by kappa and lambda. IgM shows scattered positivity. Positive for IGG4	IgG4‐related Chronic inflammation with increased plasma cells with no light chain restrictions	No surgical follow‐up	Ruling out a plasma cell proliferation disorder
II	Chronic sialadenitis	Positive for IgG4	Chronic sialadenitis Suggestive of IGG4‐related disease	No surgical follow‐up	Confirming an IgG related process
II	Chronic inflammation and macrophages in a background of acellular matrix, (mucin vs. colloid)	Positive for Thyroglobulin	Chronic inflammation and macrophages in a background of colloid	No surgical follow‐up	Identifying colloid
II	Mostly macrophages and epithelioid cells, rare atypical cells in a background of lymphocytes, crystals and cell debris	CD68 highlights macrophages; AE1/AE3 stains rare degenerated epithelial cells; Mucicarmine is negative	Mostly macrophages in a background of lymphocytes, crystals and cell debris, compatible with the clinical and radiologic impression of cystic hygroma	No surgical follow‐up	To evaluate nature of the epithelioid cells, epithelial cells versus macrophages
II	Granulomatous inflammation	GMS and Ziehl Neelsen stains are negative	Granulomatous inflammation	Necrotizing granulomatous inflammation with organisms on FITE stain, consistent with atypical mycobacterial infection	Non‐contributary
II	Polymorphous lymphocytes with atypical lymphocytes and histiocytes, an infectious process cannot be entirely excluded	Mixed population of CD3 positive T cells and CD20 positive B cells, Warthin–Starry, Brown Hopps, and GMS special stains and AFB and spirochete immunostains are negative for bacterial and fungal organisms	Reactive lymph node, no micro‐organisms identified	Rosai‐Dorfman disease	To rule out an infectious process

**TABLE 3:** MSRSGC category III: *Atypia of Undetermined significance* (*AUS*), *c*ytology diagnosis, type of ancillary studies performed on cell block, and surgical pathology diagnosis (if applicable)

MSRSGC	FNA diagnosis without ancillary studies	Ancillary studies on cell block	FNA diagnosis with ancillary studies	Surgical pathology diagnosis	Reason for ancillary studies
III	Acellular matrix (mucin vs. colloid), chronic inflammation, macrophages	Matrix positive for thyroglobulin	Colloid, chronic inflammation, macrophages	No surgical follow‐up	Identifying nature of acellular material
III	Atypical mononuclear cells suspicious for Hodgkin's disease	Negative for CD15 and CD30; Equivocal for CD68 and HAM56	Atypical mononuclear cells in background of lymphocytes	No surgical follow‐up	Rule out Hodgkin's disease
III	Rare atypical epithelial cell, chronic inflammation, a low grade mucoepidermoid carcinoma cannot be entirely excluded	Negative for mucicarmine	Rare atypical epithelial cells, chronic inflammation	No surgical follow‐up	Rule out mucoepidermoid carcinoma
III	Salivary gland lesion composed of atypical epithelial cells and necrosis	Squamous cells are positive for p63 and negative for mucicarmine	Salivary gland lesion composed of atypical squamous cells and necrosis	No surgical follow‐up	Identifying nature of the epithelial cells, squamous versus glandular
III	Rare atypical poorly preserved epithelial cells in a background of cystic changes, cellular debris, necrosis, acute inflammation, and benign acinar tissue	Squamous cells are positive for CK5/6	Rare atypical squamous cells in a background of cystic changes, cellular debris, necrosis, acute inflammation, and benign acinar tissue	No surgical follow‐up	Identifying nature of the epithelial cells
III	Atypical lymphoid cells favor reactive lymph node	CD3 and CD20 stain mixture of T‐cell and B‐cells respectively. BCL6 highlights scattered germinal centers, which are negative for BCL2. CD23 highlights follicular dendritic networks and mantle zone cells	Atypical lymphoid cells cannot exclude lymphoma	No surgical follow‐up	To differentiate reactive lymph node versus atypical lymphoid proliferation
III	Rare cells with oncocytic features admixed with inflammation, acinar cells and crystals	AE1/AE3 highlights salivary gland epithelium; mucicarmine is negative	Rare oncocytic cells with mixed inflammation, acinar cells and crystals	Oncocytic cystadenomas	To rule out intracellular mucin
III	Atypical epithelial cells with focal squamous and glandular features and focal inflammation	Squamous cells are positive for CK5/6 and p63; macrophages are negative for mucicarmine	Atypical metaplastic squamous cells, favor reactive, foamy macrophages, and focal inflammation, favored a dilated salivary duct which has undergone squamous metaplasia with reactive atypia (patient has a history of treated abscess). A low grade salivary gland neoplasm with squamous metaplasia cannot be entirely excluded.	No surgical follow‐up	To confirm the nature of squamous cells and evaluate mucin in vacuolated macrophages
III	Atypical lymphoid cells in a background of normal salivary gland parenchyma, epithelioid cells, polarizable crystalline material and amorphous debris	CD68 highlights macrophages; negative for AE1/AE3 and S100; mucicarmine is non‐contributary	Atypical lymphoid cells in a background of normal salivary gland parenchyma, histiocytes, polarizable crystalline material and amorphous debris	No surgical follow‐up	To evaluate the nature of epithelioid cells
III	Acellular eosinophilic material of uncertain origin (keratin vs. amyloid), squamous cells, chronic inflammation	Squamous cells and background keratin are positive for AE1/AE3; Congo red is negative for amyloid	Abundant eosinophilic necrotic and mummified material consistent with keratin and necrotic keratinized cells, few viable squamous cells without cytologic atypia, and macrophages present	Oncocytoma	Ruling out amyloid
III	Abundant oncocytic cells with associated blood vessels, the differential diagnosis includes a reactive lesion versus a salivary gland neoplasm versus melanoma	Positive for AE1/AE3 and CAM5.2; focally positive for SOX10, S100 and mucin stain; negative for HMB45; non‐contributary for Melan A	Oncocytic cells with blood vessels, the differential diagnosis includes mucoepidermoid carcinoma versus a reactive lesion secondary to obstruction; there are no overt features of malignancy and no evidence of melanoma	Secretory carcinoma	To identify nature of the cells, epithelial and ruling out malignant melanoma

**TABLE 4:** MSRSGC category IVa: Benign neoplasm, cytology diagnosis, type of ancillary studies performed on cell block, and surgical pathology diagnosis (if applicable)

MSRSGC	FNA diagnosis without ancillary studies	Ancillary studies on cell block	FNA diagnosis with ancillary studies	Surgical pathology diagnosis	Reason for ancillary studies
IVa	Spindle cell neoplasm favor schwannoma	Positive for S100	Schwannoma	Schwannoma	Confirm Schwannoma
IVa	Oncocytic cells and lymphocytes, favor Warthin tumor, however metastatic lung adenocarcinoma cannot be entirely excluded	Negative for TTF‐1 and Napsin A	Warthin tumor (History of lung adenocarcinoma noted)	No surgical follow‐up	Rule out metastatic lung adenocarcinoma
IVa	Spindle cell neoplasm, favor schwannoma	Positive for S100; and negative for CD68	Spindle cell neoplasm, consistent with schwannoma	No surgical follow‐up	Confirm Schwannoma
IVa	Salivary gland neoplasm of uncertain malignant potential (SUMP), favor pleomorphic adenoma, however adenoid cystic carcinoma cannot be entirely excluded	Positive for p63; negative for CD117	Myoepithelial rich pleomorphic adenoma	Pleomorphic adenoma	To rule out adenoid cystic carcinoma
IVa	Salivary gland neoplasm, favor pleomorphic adenoma	Positive for CK7; CK5/6; p63 (focal); Calponin is non‐contributary	Pleomorphic adenoma	Pleomorphic adenoma	Detecting myoepithelial cells
IVa	Salivary gland neoplasm of uncertain malignant potential (SUMP) with oncocytic features and rare lymphocytes, favor Warthin tumor, however a malignant neoplasm such as acinic cell carcinoma cannot be entirely excluded	Oncocytes are negative for PAX‐8 and DOG‐1; p63 highlights basal cells	Benign salivary gland neoplasm with oncocytic features with rare lymphocytes, consistent with Warthin tumor	No surgical follow‐up	Ruling out carcinoma such as acinic cell carcinoma

**TABLE 5:** MSRSGC category IVb: Salivary gland neoplasm of uncertain malignant potential (SUMP), cytology diagnosis, type of ancillary studies performed on cell block, and surgical pathology diagnosis (if applicable)

MSRSGC	FNA diagnosis without ancillary studies	Ancillary studies on cell block	FNA diagnosis with ancillary studies	Surgical pathology diagnosis	Reason for ancillary studies
IVb	Fragments of basaloid epithelium, differential diagnosis include a salivary gland neoplasm with basaloid features versus metastatic papillary thyroid carcinoma	Negative for thyroglobulin	Salivary gland neoplasm with basaloid features	No surgical follow‐up	Rule out metastatic papillary thyroid carcinoma (History of papillary thyroid carcinoma)
IVb	Salivary gland neoplasm with focal squamous and glandular features	Positive for mucicarmine	Salivary gland neoplasm with focal squamous and mucinous features	High grade adenocarcinoma, consistent with salivary duct carcinoma	Confirming intracytoplasmic mucin
IVb	Atypical epithelial cells, cannot exclude a low‐grade salivary gland neoplasm	Positive for mucicarmine	Salivary gland neoplasm, low grade	Mucoepidermoid carcinoma	Confirming intracytoplasmic mucin
IVb	Salivary gland neoplasm	Epithelial cells labeling with AE1/AE3 and myoepithelial labeling with P63 and SMA	Biphasic salivary gland neoplasm with epithelial and myoepithelial components	No surgical follow‐up	Confirming epithelial and myoepithelial components
IVb	Low grade salivary gland neoplasm with oncocytic features, however a secondary neoplasm such as melanoma cannot be entirely excluded	Negative for S100	Low grade salivary gland neoplasm with oncocytic features	No surgical follow‐up	Rule out melanoma
IVb	Salivary gland neoplasm with basaloid features	Positive for cytokeratin; negative for CD45	Salivary gland neoplasm with basaloid features	No surgical follow‐up	Confirming the presence of epithelial cells
IVb	Salivary gland neoplasm of uncertain malignant potential	P63 highlights myoepithelial cells	Salivary gland neoplasm with prominent myoepithelial cell population and scant stroma	No surgical follow‐up	Identifying nature of the neoplastic cells
IVb	Neoplasm with spindled and histiocytoid cells	Rare cells positive for S100; non‐contributary MNF116; and HMB45	Granular/histiocytoid neoplasm. The cytomorphologic differential diagnosis includes granular cell tumor, schwannoma and PEComa. Although this lesion is favored to be benign, a low‐grade salivary gland neoplasm with oncocytic features, such as acinic cell carcinoma, cannot be completely ruled out	Granular cell tumor	To identify nature of the neoplastic cells
IVb	Atypical spindle cell neoplasm	Negative for CK5/6; p63, and S100, Ki‐67 less than 25%	Atypical spindle cell neoplasm, with focal basaloid epithelioid groups of uncertain significance in a myxoid background. These spindle cells may therefore not be myoepithelial, but only scant tissue is present for assessment. The findings are concerning for a malignant neoplasm, such as a low‐grade sarcoma, but the spindle cells are not unequivocal for malignancy and the differential diagnosis includes a spectrum of tumors.	Myofibroblastic sarcoma	To identify nature of the neoplastic cells
IVb	Neoplasm with basaloid features	Positive for P63; negative for CD31; CD34 and FLI‐1	Salivary gland neoplasm with basaloid features. The differential diagnosis includes recurrence of the patient's prior basal cell carcinoma and primary salivary gland neoplasms (basal cell adenoma, basaloid squamous cell carcinoma, and adenoid cystic carcinoma)	No surgical follow‐up	To identify nature of the neoplastic cells
IVb	Salivary gland neoplasm with basaloid features	p63 highlights myoepithelial cells; negative for c‐KIT	Salivary gland neoplasm with basaloid features. The differential diagnosis includes basal cell adenoma and pleomorphic adenoma; however, other low‐grade basaloid neoplasms should also be considered in the differential. Lack of C‐kit expression does not favor the possibility of adenoid cystic carcinoma to be considered in the differential.	Basal cell adenoma	To identify nature of the neoplastic cells
IVb	Neoplasm with clear cells features	Positive for AE1/AE and CD10; focally positive for p63 and Calponin; negative for S100; DOG‐1; c‐KIT; RCC and PAX‐8	Neoplasm with clear cells features, Although CD10 positivity raises concern for metastatic renal cell carcinoma, the fact that the neoplastic cells are negative for RCC and PAX8 makes this possibility less likely although not entirely ruled out. CD10, is also a myoepithelial marker and together with focal positivity for p63 and calponin raises the possibility of a primary salivary gland neoplasm of epithelial‐myoepithelial origin	Metastatic renal cell carcinoma	To rule out acinic cell carcinoma and metastatic renal cell carcinoma in a patient with history of kidney malignancy status post nephrectomy
IVb	Biphasic neoplasm with cytologic atypia, suspicious for malignancy	Positive for p63 and CK5/6; negative for S100 and mucicarmine	Biphasic neoplasm with cytologic atypia. The neoplasm shows epithelioid areas as well as spindled cells with some admixed matrix. The tumor is markedly cellular and has areas with prominent cytologic atypia. Focal areas of squamous differentiation are also seen. The differential diagnosis includes pleomorphic adenoma with atypia, carcinoma ex pleomorphic adenoma, basal cell adenoma and mucoepidermoid carcinoma.	Pleomorphic adenoma	To evaluate nature of the neoplastic cells
IVb	Salivary gland neoplasm with oncocytes, lymphocytes, few squamous cells and debris	Positive for p63 and CK5/6; negative for mucicarmine	Low grade salivary gland neoplasm with oncocytes, lymphocytes, few squamous cells and debris. The differential diagnosis includes a Warthin tumor with squamous differentiation versus a low grade mucoepidermoid carcinoma with oncocytic change	Warthin tumor	To evaluate nature of the neoplastic cells
IVb	Low grade salivary gland neoplasm	Positive for AE1/AE3; negative for S100; Synaptophysin; chromogranin; and DOG‐1	Low grade salivary gland neoplasm	No surgical follow‐up	To evaluate nature of the neoplastic cells
IVb	Salivary gland neoplasm with basaloid features	Negative for c‐KIT	Myoepithelial rich salivary gland neoplasm. Based on morphology a diagnosis of cellular pleomorphic adenoma is favored. The other lesions to consider in the differential include monomorphic adenoma and myoepithelioma. Adenoid cystic carcinoma is less likely due to negative c‐Kit stain.	Myoepithelioma	To rule out adenoid cystic carcinoma
IVb	Low grade salivary gland neoplasm with focal squamous features and necrosis	Positive for S100 and p63; negative for c‐KIT and mucicarmine	Low grade salivary gland neoplasm with focal squamous features and necrosis. The differential diagnosis includes epithelial‐myoepithelial salivary gland neoplasm including pleomorphic adenoma, epithelial/myoepithelial carcinoma and low grade mucoepidermoid carcinoma	Pleomorphic adenoma	To identify nature of the neoplastic cells
IVb	Salivary gland neoplasm	Negative for c‐KIT	Salivary gland neoplasm, favor cellular pleomorphic adenoma. While a cellular pleomorphic adenoma or myoepithelioma is favored, a low‐grade malignancy including myoepithelial carcinoma cannot be entirely excluded	Cellular pleomorphic adenoma	To rule out adenoid cystic carcinoma
IVb	Neoplasm with focal clear cell features	positive for AE1/AE3, p63, EMA (weak, focal), SMA, SMM‐HC (weak), negative for desmin, S100, CD31 and HMB‐45	Neoplasm with focal clear cell features, favor pleomorphic adenoma, however a metastatic process cannot be excluded	Cellular pleomorphic adenoma	To identify nature of the neoplastic cells
IVb	Salivary gland neoplasm	Negative for cytokeratin AE1/AE3, SMA, HMB‐45 and Melan A and focally positive for S100	Salivary gland neoplasm with abundant myoepithelial cells	Myoepithelioma	To identify the nature of cells
IVb	Low grade salivary gland neoplasm	Positive for S100; negative for DOG‐1, mammaglobin, and mucicarmine	Low grade salivary gland neoplasm, with eosinophilic vacuolated cytoplasm on cell block	Secretory carcinoma	To identify nature of the neoplastic cells
IVb	Low grade salivary gland neoplasm, favor pleomorphic adenoma	Neoplastic epithelial cells positive for AE1/3 and C‐KIT; Neoplastic myoepithelial cells positive for p63, AE1/3, S100, and calponin; Negative for synaptophysin	Low grade salivary gland neoplasm, favor pleomorphic adenoma	Recurrent esthesioneuroblastoma	To identify nature of the neoplastic cells
IVb	Cellular epithelial neoplasm of salivary gland origin	Negative for mucicarmine	Cellular epithelial neoplasm of salivary gland origin	Metastatic carcinoma with neuroendocrine differentiation	Rule out mucoepidermoid carcinoma

**TABLE 6:** MSRSGC category IV: Suspicious for malignancy, cytology diagnosis, type of ancillary studies performed on cell block, and surgical pathology diagnosis (if applicable)

MSRSGC	FNA diagnosis without ancillary studies	Ancillary studies on cell block	FNA diagnosis with ancillary studies	Surgical pathology diagnosis	Reason for ancillary studies
V	Suspicious for malignant neoplasm	S100; HMB45; AE1/AE3, and CAM5.2 non‐contributary due to limited cells	Suspicious for malignant neoplasm	Salivary duct carcinoma	To identify nature of the neoplastic cells
V	Suspicious for malignant neoplasm	Positive for AE1/AE3 and CAM5.2	Suspicious for malignant neoplasm. The differential diagnosis includes acinic cell carcinoma and secretory carcinoma.	Salivary duct carcinoma	To identify nature of the neoplastic cells
V	Atypical lymphoid infiltrate suspicious for lymphoproliferative disorder	Positive CD20 B cell lymphocytes; scattered CD3 positive T cells	Atypical lymphoid infiltrate suspicious for lymphoproliferative disorder	MALT lymphoma	To identify nature of the lymphocytes
V	Suspicious for secretory carcinoma	Positive for S100; negative for DOG‐1, mammaglobin, and mucicarmine	Suspicious for secretory carcinoma	Secretory carcinoma	To identify nature of the neoplastic cells

**TABLE 7:** MSRSGC category IV: Malignant cytology diagnosis, type of ancillary studies performed on cell block, and surgical pathology diagnosis (if applicable)

MSRSGC	FNA diagnosis without ancillary studies	Ancillary studies on cell block	FNA diagnosis with ancillary studies	Surgical pathology diagnosis	Reason for ancillary studies
VI	Malignant neoplasm, favor carcinoma	Positive for CK‐7 and Mammaglobin Negative for CK20; p63; CK5/6; p40; S100; HMB45; TTF‐1; Napsin A; Thyroglobulin; CDX2 and Mucicarmine	Malignant neoplasm, favor salivary duct carcinoma	Salivary duct carcinoma	To identify nature of the neoplastic cells
VI	Neoplasm with neuroendocrine features	Positive for Synaptophysin and chromogranin Negative for Actin	Metastatic neuroendocrine tumor	No surgical follow‐up	To identify nature of the neoplastic cells
VI	Adenocarcinoma	Positive for CK7; negative for CK20	Adenocarcinoma	Salivary duct carcinoma	To identify nature of the neoplastic cells
VI	Malignant neoplasm favor Metastatic melanoma	Positive for HMB 45	Metastatic malignant melanoma	Metastatic malignant melanoma	Confirming metastatic melanoma (history of melanoma)
VI	Poorly differentiated non‐small cell carcinoma	Negative for thyroglobulin	Poorly differentiated non‐small cell carcinoma. Negative for papillary thyroid carcinoma	Acinic cell carcinoma	Ruling out papillary thyroid carcinoma (History of papillary thyroid carcinoma)
VI	Mucoepidermoid carcinoma, low grade	Positive mucicarmine stain	Mucoepidermoid carcinoma, low grade	No surgical follow‐up	Detecting mucin
VI	Mucoepidermoid carcinoma	Positive mucicarmine stain	Mucoepidermoid carcinoma	Mucoepidermoid carcinoma	Detecting mucin
VI	Malignant neoplasm	Positive for CD56, chromogranin, synaptophysin (weakly and focally), and CD56. negative for AE1/AE3, CD45, S100, p63, L‐Actin, actin, MGN, and CD45	Malignant neoplasm, favor metastatic oligodendroglioma	Metastatic oligodendroglioma	To confirm metastatic oligodendroglioma (history of oligodendroglioma)
VI	Suspicious for lymphoma	Positive for CD20; Negative for CD10, BCL‐6, CD5, CD23, and cyclin D1	MALT lymphoma	No surgical follow‐up	Confirming the diagnosis
VI	High grade neoplasm, favor carcinoma	Positive for cytokeratin; Negative for chromogranin, calcitonin, s‐100, HMB‐45, thyroglobulin, mucin	High grade carcinoma	High grade adenocarcinoma	Confirming the diagnosis of carcinoma
VI	Malignant neoplasm with small cell features	Positive for CD56, chromogranin, and synaptophysin; negative for O13, CK20, CD3, CD10, CD20, CD45, Kappa and Lambda light chains; equivocal for AE1/AE3	Small cell carcinoma	No surgical follow‐up	Confirming the diagnosis
VI	Acinic cell carcinoma	Positive for pan cytokeratin; Negative for GFAP, S100, smooth muscle actin, and mucicarmine	Acinic cell carcinoma	No surgical follow up	Confirming the diagnosis
VI	Squamous cell carcinoma	Positive for P16 and HPV	HPV‐related Squamous cell carcinoma	HPV‐related squamous cell carcinoma	Detection of high‐risk HPV
VI	Involved by multiple myeloma	Positive for CD138 and kappa; negative for lambda	Involved by multiple myeloma, kappa light chain restricted	No surgical follow‐up	Confirming multiple myloma (history of multiple myeloma)
VI	Metastatic papillary thyroid carcinoma	Positive for thyroglobulin	Metastatic papillary thyroid carcinoma	No surgical follow‐up	Confirming Metastatic papillary thyroid carcinoma (history of papillary thyroid carcinoma)
VI	Poorly differentiated malignant neoplasm	Positive for cytokeratin, AE1/AE3, CAM5.2, and mucicarmine; negative for S100, HMB45, and Melan A	Poorly differentiated adenocarcinoma	Invasive salivary duct carcinoma	To differentiate carcinoma from melanoma
VI	Metastatic squamous cell carcinoma	Positive for P63 and CAM5.2	Metastatic squamous cell carcinoma	Poorly differentiated squamous cell carcinoma	To confirm the diagnosis
VI	Squamous cell carcinoma	Positive for p16 and HPV ISH	HPV‐related Squamous cell carcinoma	No surgical follow‐up	Detection of high‐risk HPV
VI	Suspicious for large B cell lymphoma	CD20 stains confluent sheets of large B; dimly positive for BCL‐2, and lack CD5 and CD10. Ki‐67 of 50%–60%. CD23, NKX3.1 are negative.	Large B cell lymphoma	No surgical follow‐up	Confirming the diagnosis
VI	Poorly differentiated carcinoma with squamous features	Positive for p40	Poorly differentiated squamous cell carcinoma	Invasive poorly differentiated squamous cell carcinoma	Confirming the diagnosis
VI	High grade carcinoma	Positive for AR; negative for S100; Mammaglobin; HER2/Neu; and mucin stain	High grade carcinoma, favor salivary duct carcinoma	Salivary duct carcinoma, micropapillary pattern	Confirming the diagnosis
VI	Poorly differentiated malignant neoplasm	Positive for AE1/AE3; p63 and p40; negative for SOX10; MART‐1; Melan A; S100; CK7; CK20; c‐KIT; p16; Mucin stain	Poorly differentiated squamous cell carcinoma	Poorly differentiated squamous cell carcinoma	To evaluate nature of the neoplastic cells
VI	Salivary gland neoplasm, most consistent with secretory carcinoma	Positive for CK19; Mammaglobin; and S100; negative for p63; DOG‐1, and PAS‐D	Salivary gland neoplasm, most consistent with secretory carcinoma	No surgical follow‐up	Confirming the diagnosis
VI	Suspicious for secretory carcinoma	Positive for CK7; CK8/18; SMA; Mammaglobin; and S100; negative for CK20; Ber‐Ep4; CK5/6 and p63	Secretory carcinoma	No surgical follow‐up	Confirming the diagnosis
VI	Low grade neoplasm. The differential diagnosis includes acinic cell carcinoma and less likely a metastatic process	Positive for CK7 and Vimentin; negative for CK20; P63 and CD10	Acinic cell carcinoma	No surgical follow‐up	To exclude metastatic carcinoma
VI	Malignant neoplasm, favor sarcoma	Positive for Myogenin; MyoD1; desmin; and SMA	Alveolar rhabdomyosarcoma	No surgical follow‐up	To confirm the diagnosis
VI	Metastatic melanoma	Positive for S100	Metastatic melanoma	No surgical follow‐up	To confirm the diagnosis
VI	Poorly differentiated neoplasm with necrosis, suggestive of metastatic glioblastoma	Positive for GFAP; focally positive for AE1/AE3; negative for S100	Poorly differentiated neoplasm with necrosis, consistent with metastatic glioblastoma	No surgical follow‐up	To confirm the diagnosis
VI	Poorly differentiated malignant neoplasm with necrosis	Positive for AE1/AE3 and CK7; focally positive for GCDFP; negative for Melan A; S100; CDX‐2; TTF‐1; Mucin stain	Poorly differentiated carcinoma with necrosis	No surgical follow‐up	To evaluate nature of the neoplastic cells and rule out a metastatic process
VI	Squamous cell carcinoma	Negative for p16; HPV ISH	Squamous cell carcinoma, non‐HPV related	No surgical follow‐up	Rule out HPV
VI	Suspicious for Large B cell lymphoma	Positive for CD20; CD10 and BCL‐6; negative for BCL‐2; CD45; CD30; MUM1; EBV ISH, few T cells positive for CD3; CD43; BCL‐2	Large B cell lymphoma	No surgical follow‐up	Confirming large B cell lymphoma
VI	Malignant neoplasm suspicious for Metastatic Merkel cell carcinoma	Positive for synaptophysin and CK20; negative for chromogranin	Metastatic Merkel cell carcinoma	Metastatic Merkel cell carcinoma	Confirming the diagnosis
VI	Suspicious for mucoepidermoid carcinoma	Positive PAS stain	Mucoepidermoid carcinoma	Mucoepidermoid carcinoma	Detection of mucin
VI	Squamous cell carcinoma	Positive for p16; negative for Mucin stain	p16‐positive Squamous cell carcinoma	No surgical follow‐up	To evaluate p16 and detect mucin
VI	Squamous cell carcinoma	Positive for p40 and p16	p16‐positive Squamous cell carcinoma	No surgical follow‐up	To confirm the diagnosis and detect p16
VI	Squamous cell carcinoma	p16 positive	p16‐positive Squamous cell carcinoma	No surgical follow‐up	To detect p16
VI	Atypical lymphoid cells concerning for Hodgkin lymphoma	Positive for CD30 and CD15	Hodgkin lymphoma	Classical Hodgkin lymphoma type, EBV+, recurrent, post‐transplant	To confirm a diagnosis
VI	Carcinoma	AR equivocal; negative for TTF‐1 and NapsinA	Salivary duct carcinoma	Salivary duct carcinoma	To confirm a diagnosis and rule out a metastatic process in a patient with history of lung adenocarcinoma
VI	Atypical lymphoid tissue	Positive for CD45, CD20, and vimentin, negative for CD30; AE1/AE3; CAM5.2; and S100	Atypical lymphoid tissue consistent with lymphoma	Follicular lymphoma, grade 3B	To evaluate nature of the neoplastic cells
VI	Carcinoma	Positive for AE1/AE3 and AR; focally positive for mammaglobin and p63; negative for S100	Salivary duct carcinoma	No surgical follow‐up	To confirm a diagnosis
VI	Salivary gland neoplasm with features suggestive of acinic cell carcinoma	Positive for DOG‐1; negative for mammaglobin; p63 and S100	Acinic cell carcinoma	Acinic cell carcinoma	To confirm a definitive diagnosis
VI	Poorly differentiated carcinoma	Focally positive for P40; CK5/6	Poorly differentiated squamous cell carcinoma	Poorly differentiated squamous cell carcinoma	Confirming the diagnosis
VI	Poorly differentiated malignant neoplasm	Positive for CAM5.2 Negative for CK20; chromogranin; synaptophysin; TTF‐1; CD20 and CD5	Poorly differentiated carcinoma	No surgical follow‐up	To evaluate nature of the neoplastic cells
VI	Malignant salivary gland neoplasm	Negative for AR and HER2/Neu	Malignant salivary gland neoplasm	Salivary Duct carcinoma	To confirm a diagnosis
VI	Poorly differentiated malignant neoplasm	Positive for AE1/AE3	Poorly differentiated carcinoma	No surgical follow‐up	To confirm carcinoma
VI	Poorly differentiated carcinoma with squamous differentiation	Positive for AE1/AE3; CK19; p63; EGFR; focally positive for GATA3; negative for AR and mucicarmine	Poorly differentiated carcinoma with squamous differentiation	Metastatic squamous cell carcinoma	To confirm carcinoma and squamous differentiation
VI	Neoplasm with spindled and epithelioid cells present	Positive for AE1/AE3; vimentin; S100 and CD10; negative for RCC; CK7; SMA; TTF1	Neoplasm with spindled and epithelioid cells present	No surgical follow‐up	To evaluate nature of the neoplastic cells and rule out a metastatic process
VI	Malignant neoplasm suggestive of Merkel cell carcinoma	Positive for AE1/AE3; CK20 and PAX5	Merkel cell carcinoma	No surgical follow‐up	To confirm metastasis of patient's known Merkel cell carcinoma
VI	Poorly differentiated malignant neoplasm	Positive for AE1/AE3; negative for S100	Poorly differentiated malignant neoplasm, favor carcinoma	Salivary duct adenocarcinoma with in situ component	To confirm carcinoma
VI	Poorly differentiated malignant neoplasm	Positive for myogenin, desmin, and AE1/AE3; negative for CAM5.2	Poorly differentiated malignant neoplasm consistent with patient's known malignant neoplasm	No surgical follow‐up	To confirm recurrence or metastasis of patient's known malignant neoplasm
VI	Poorly differentiated malignant neoplasm with spindle and epitheloid features	positive for p63 and CAM5.2 (weak focal); negative for AE1/AE3, S100, HMB45, MiTF and Melan‐A	Poorly differentiated malignant neoplasm with spindle and epitheloid features	Metastatic melanoma	To evaluate nature of the neoplastic cells
VI	Poorly differentiated squamous cell carcinoma	Negative for p16	Poorly differentiated squamous cell carcinoma, P16 negative	Metastatic squamous cell carcinoma	To exclude HPV related carcinoma
VI	Poorly differentiated malignant neoplasm	Positive for AE1/AE3; negative for S100; Melan A	Poorly differentiated carcinoma	No surgical follow‐up	To confirm carcinoma and ruling out melanoma
VI	Poorly differentiated carcinoma with neuroendocrine features	Positive for AE1/AE3; CK20; chromogranin and synaptophysin; negative for CK7; S100; HMB45; Melan‐A and TTF‐1	Poorly differentiated carcinoma with neuroendocrine features	Metastatic poorly differentiated carcinoma	To confirm carcinoma and ruling out melanoma
VI	Metastatic melanoma	Positive for Melan A and HMB45, negative for AE1/AE3 and S100	Metastatic melanoma	Melanoma	To confirm metastatic melanoma
VI	Metastatic melanoma	Positive for S100; HMB45; Melan A; negative for AE1/AE3	Metastatic melanoma	Melanoma	To confirm metastatic melanoma
VI	Poorly differentiated carcinoma with vacuolated and pleomorphic cells	Positive for AE1/AE3, CK20 Negative for S100; HMB45; TTF1, and mucicarmine	Poorly differentiated carcinoma with vacuolated and pleomorphic cells	Poorly differentiated carcinoma	To confirm diagnosis of carcinoma and excluding a metastatic process
VI	Melanoma	Positive for Melan A; non‐contributary for S100, AE1/AE3, HMB45	Melanoma	Melanoma	To confirm metastatic melanoma
VI	Squamous cell carcinoma	Negative for p16	Squamous cell carcinoma, p16 negative	Metastatic squamous cell carcinoma	Excluding HPV related carcinoma
VI	Poorly differentiated carcinoma with neuroendocrine features	Positive for CAM5.2; synaptophysin and chromogranin	Poorly differentiated carcinoma with neuroendocrine features	Metastatic neuroendocrine carcinoma from the patients known sinonasal primary	To confirm the diagnosis

Abbreviations: Ca, carcinoma; IHC, immunohistochemical stains; ISH, in situ hybridization; MALT, marginal zone B‐cell lymphoma of mucosa‐associated lymphoid tissue; SI, surgical intervention.

Surgical follow‐up was available in 59 cases (50.4%), ranging from 27% to 100% of cases within each MSRSGC category. Ancillary studies were helpful in 60%–100% of cases in each MSRSGC category. Two out of three (66.6%) cases in category I had surgical follow up and both were diagnosed malignant (cystic mucoepidermoid carcinoma and chronic lymphocytic leukemia (CLL). Three out of ten cases (30%) in category II had surgical follow up, one was diagnosed as benign neoplasm (Rosai‐Dorfman disease) and two inflammatory/reactive (benign lymph node with follicular hyperplasia and necrotizing granulomatous inflammation). In category III, 3 out of 10 cases had surgical follow up. One case was diagnosed malignant (secretory carcinoma) and two were benign neoplasm (oncocytic cystadenoma and oncocytoma). In category IVa, 3 out of 6 cases had surgical follow up and they were diagnosed benign neoplasm (two pleomorphic adenomas and one schwannoma). In category IVb, 16 out of 23 cases had surgical follow up including 7 malignant cases (one of each high‐grade adenocarcinoma, mucoepidermoid carcinoma, myofibroblast sarcoma, metastatic renal cell carcinoma, secretory carcinoma, esthesioneuroblastoma, metastatic carcinoma with neuroendocrine differentiation) and 9 benign cases (4 pleomorphic adenomas, 2 myoepitheliomas, one granular cell tumor, one basal cell adenoma, and one Warthin tumor). All four category V cases were malignant on surgical follow‐ up (two salivary duct carcinomas, one secretory carcinoma, and one MALT [Mucosa‐associated lymphoid tissue] lymphoma). In category VI, 28 out of 60 cases were confirmed malignant on surgical follow up (7 salivary duct carcinomas, 7 squamous‐cell carcinomas including one HPV‐related case, 5 metastatic melanomas, 2 acinic cell carcinomas, 2 mucoepidermoid carcinomas, 2 lymphoma cases; one Hodgkin disease and one follicular lymphoma, and one of each recurrent oligodendroglioma, high grade adenocarcinoma, and Merkel cell carcinoma). ROM was 100% in both the suspicious for malignancy (V), and malignant (VI) categories. A non‐neoplastic (II) case representing reactive lymph node on FNA with clusters of histiocytes was diagnosed as Rosai‐Dorfman disease on surgical follow‐up.

## 4 DISCUSSION

In this study we evaluated the utility of ancillary studies including IHC and histochemical staining, and ISH performed on FNA cell blocks in diagnosis of salivary gland lesions classified according to MSRSGC, and the impact on clinical decision‐making. Ancillary studies applied on SG FNA such as molecular studies, or FISH were not included in this study. The low number of cases in this study is an evidence that cell blocks either are not routinely prepared for all SG FNA cases or they may not contain sufficient material for subsequent studies. Therefore, this study and its finding presents a small number of cases that contained sufficient material for ancillary studies.

The amorphous matrix in the background posed diagnostic difficulties, particularly in cystic and hypocellular specimens. Mucicarmine stain and thyroglobulin were used to highlight mucin and colloid in two cystic cases, respectively (Table 2).

The presence of inflammatory cells, epithelioid histocytes and granulomatous inflammation triggered the initial pathologists to investigate an underlying infectious process. Gram stain, GMS stain, Zeihl Neelsen stain, Warthin–Starry stain, Brown Hopps stain, and spirochete immunostains were utilized in these cases. Although a negative stain cannot exclude an infectious process, a positive stain detecting microorganisms confirms an infectious process. These stains were utilized more often in non‐neoplastic cases (Tables 1 and 2). The presence of atypical lymphocytes on aspirated material can be due to either reactive changes or a lymphoproliferative disorder. Flow cytometry studies can be requested on aspirated material if there is on‐site evaluation for specimen adequacy. Immunostains used for detection of lymphoproliferative/hematopoietic disorders such as CD3, and CD20 were commonly utilized to rule out monoclonal proliferation of T cell or B cell lymphocytes, respectively. A selective panel of immunostains along with cytomorphology confirmed the diagnosis of lymphoma in several cases (Table 7). Recurrence of Hodgkin lymphoma was confirmed by positive CD15 and CD30 immunostains in a post‐transplant patient. Plasma cell markers such as CD138, kappa and lambda were used to differentiate polyclonal from monoclonal plasma cell proliferations. These diagnostic or confirmatory immunostains for detection of lymphoproliferative or hematopoietic disorders improved the MSRSGC by separating malignant cases from reactive cases and decreasing the number of cases in indeterminate categories (atypical or suspicious). The sampling issue was a contributing factor to cytology diagnosis of indeterminate category in a subset of cases. In cyst content cases with epithelioid, poorly preserved or atypical macrophages, histiocytic markers, such as CD68, can confirm their identity and help prevent an atypical diagnosis. IgG‐related sialadenitis was diagnosed in a few cases by applying IgG4 immunostain in those cases that were suspicious for IgG4‐related chronic sialadenitis.

Clinical history of prior malignancy along with cytomorphologic features suspicious for a recurrence or a metastatic process played a key role in selecting immunostains in a subset of patients. For example, cytokeratin AE1/AE3, CK20, and PAX5 immunostains were ordered on aspirated material from parotid gland of a patient previously diagnosed for Merkel cell carcinoma to confirm a metastatic process or TTF‐1 and Napsin‐A were reviewed to rule out a metastatic lung adenocarcinoma. The material in cell block of cases with confirmed recurrence or metastatic disease can be further utilized for molecular testing, which can be explored in a future study. Additionally, p16 and HPV in situ hybridization were utilized to detect HPV‐related or p16 positive squamous‐cell carcinoma cases, which has prognostic implication compared to its HPV‐negative or p16‐negative counterpart.

Immunostains and mucicarmine stain were used to confirm a diagnosis of a salivary gland neoplasm in a subset of cases. For instance DOG‐1 was used to confirm a case of acinic cell carcinoma.^11^ Mammaglobin was helpful in diagnosis of secretory carcinoma cases.^12^ Androgen receptor immunostain was positive in salivary duct carcinoma, while p63 was negative.^13^, ^14^ However, unusual or uncommon cytomorphologic findings of salivary gland neoplasms guided the pathologists to select immunostains based on those findings. In cases with atypical or poorly‐preserved epithelial fragments, p40 and p63 highlighted squamous differentiation. Cytokeratin AE1/AE3 and CAM5.2 confirmed the epithelial origin of neoplastic cells in several cases. Pleomorphic adenoma is the most common salivary gland neoplasm, which is usually diagnosed on routine stains. However, immunostains and mucicarmine stain were utilized in several cases of pleomorphic adenomas due to their unusual cytomorphologic presentations such as focal clear cell features or necrosis. Myoepithelial cells of pleomorphic adenomas can create diagnostic challenges when present in high proportion of cells or when presenting with variable morphology such as spindle cell morphology. Cellular pleomorphic adenomas presented with basaloid features were evaluated with p63 and c‐KIT markers to rule out adenoid cystic carcinoma. Myoepithelial cells in pleomorphic adenoma are immunoreactive for p40 and p63 and negative for c‐KIT, while adenoid cystic carcinoma is immunoreactive for c‐KIT and negative for p40 and p63.^15^ Mucicarmine stain was used to evaluate cells with intracellular mucin such as those seen in mucoepidermoid carcinoma cases. Metaplastic changes associated with necrosis or atypia raised the possibility of a malignant process in several cases otherwise appearing benign. Squamous metaplasia presented as necrotic keratinized cells and keratin as abundant eosinophilic necrotic and mummified material, which were confirmed by AE1/AE3 and amyloid stain in a case of oncocytoma. Squamous metaplasia and numerous foamy macrophages in a Warthin tumor raised the possibility of a low grade mucoepidermoid carcinoma. Mucin stain was negative and p63 highlighted squamous cells. Of note, all cases with unusual presentations which were accompanied with ancillary studies, were reviewed by another cytopathologist with expertise in salivary gland cytology in all three institutions. Based on these findings, it is evident that ancillary studies may reduce or refine the number of atypical diagnoses to more definitive diagnostic categories of MSRSGC.

## 5 CONCLUSION

This multi‐institutional study demonstrates the diagnostic utility of ancillary studies including immunohistochemistry, histochemistry, in situ hybridization, and stains for infectious agents in cell blocks prepared from aspirated salivary gland lesions in a very small subset of cases. Ancillary studies performed on cell blocks assisted to further characterize: 1) the atypical lymphocytes, neoplastic cells or their origin, 2) the matrix in the background (mucin vs. colloid), 3) unusual presentation of neoplasms and metaplastic changes, and 4) to rule out a metastatic process of a known malignancy. Ancillary studies performed on SG FNA cell blocks with sufficient material can improve the diagnostic yield by further characterization of the atypical/neoplastic cells, particularly in MSRSGC categories IVa–VI. Ancillary studies should be used judiciously and case‐based to improve diagnosis in challenging cases. The findings of this study are more case‐based and future studies with larger cohorts are required to evaluate the comprehensive role of ancillary studies, including molecular studies and FISH on cell blocks, prepared from SG FNA specimens.

## CONFLICT OF INTEREST

No conflict of interest declared.

## AUTHOR CONTRIBUTIONS

*Background research, drafting of the manuscript, conception of the idea, and critical revision*: Seena Tabibi. *Background research, drafting of the manuscript, conception of the idea, and critical revision*: Matthew Gabrielson. *Background research, drafting of the manuscript, conception of the idea, and critical revision*: Carla Saoud. *Background research, drafting of the manuscript, conception of the idea, and critical revision*: Katelynn Davis. *Background research, drafting of the manuscript, conception of the idea, and critical revision*: Sintawat Wangsiricharoen. *Data collection and editing*: Ryan Lu. *Data collection and editing*: Isabella Tondi Resta. *Data collection, and critical revision of the manuscript for important intellectual content*: Kartik Viswanathan. *Collation of cases and critical revision of the manuscript for important intellectual content*: William C. Faquin. *Collation of cases and critical revision of the manuscript for important intellectual content*: Zubair Baloch. *Conception of the idea for the manuscript and its design and coordination, collation of cases, visualization and critical revision of the manuscript for important intellectual content*: Zahra Maleki. All authors have read and approved the final manuscript and have declared that they qualify for authorship.

## Data Availability

The data will be available upon request.
